# Swedish women reaching post-traumatic growth after an intimate partner violence relationship: A study of formal help and growth after trauma

**DOI:** 10.1177/14034948231222366

**Published:** 2024-01-07

**Authors:** Olivia Claeson, Mikaela Rydén Ragnar, Agnes Åström, Yunhwan Kim

**Affiliations:** Department of Psychology, Lund University, Sweden

**Keywords:** Post-traumatic growth, intimate partner violence, formal help, women, Sweden

## Abstract

**Aims:**

This study examined whether women in Sweden who had been in an intimate partner violence (IPV) relationship reached post-traumatic growth (PTG) and if the level of PTG differed for women who had received formal help compared with women who had not received formal help. The study also examined whether there was a difference in the level of PTG depending on which type of formal help the women had received.

**Methods:**

The data were collected through an online self-report survey. A total of 166 women took part in the study.

**Results:**

The results showed that 69.3% (*n* = 115) of the women reported a pre-determined or higher level of PTG attainment and that those who had received formal help reported a higher level of PTG than those who had not received formal help. There was no difference in the level of PTG depending on which type of formal help the women had received.

**Conclusions:**

The majority of the Swedish women in this study who had lived in an IPV relationship attained PTG. Although formal help appeared to help the women attain PTG, the type of the formal help did not seem to have a crucial role in attaining different levels of PTG. These findings are discussed in the light of the future research directions and public health measures to better support women who experience IPV.

## Introduction

The World Health Organization [1] describes intimate partner violence (IPV) as physical, sexual and emotional (psychological) abuse and controlling behaviour conducted by an intimate partner. Sweden has one of the highest reported frequencies of IPV among European Union countries [2]. One Swedish study reported that 28% of the participating women had been exposed to at least one form of IPV in the previous year [3]. Women who have experienced IPV often suffer from anxiety, post-traumatic stress disorder and other mental illnesses [4–6], which is a global public health problem [7].

Research on IPV has primarily focused on the negative consequences after trauma; however, research later redirected its focus to the possibility of positive development following trauma [8–10]. Post-traumatic growth (PTG) describes how someone not only recovers after trauma, but how they can also develop a positive psychological change and progress further than before [10]. PTG includes five factors: personal strength, relating to others, spiritual change, new possibilities and appreciation of life [9]. A few studies have indicated that women who experience IPV can reach PTG [11,12]; however, to date, no empirical investigation has carried out made in Sweden.

Previous studies have shown that different types of formal help – such as help from therapists, women’s shelters, social workers, counsellors, leaders within religious communities and group therapy – can contribute to the development of PTG in women who have experienced IPV [12–25]. No study of IPV in Sweden has, to our knowledge, investigated formal help. Furthermore, no study, internationally, has compared (a) attainment and the level of PTG between women who have received formal help and women who have not and (b) the level of PTG depending on which type of formal help a woman has received. To fill these research gaps, this study investigated whether women in Sweden who experience IPV reach PTG, if the level of PTG differs between those who receive formal help after IPV and those who do not, and between those who receive different types of formal help.

## Method

### Sample

The sample (*N* = 166) were people who answered ‘yes’ to the question ‘I identify myself as a woman’, were over 18 years old, had been in an IPV relationship and who provided their consent to participate in the study. They were recruited mainly through the social media platforms of 21 Swedish women’s shelters. The survey was also shared on our private social media platforms and three Facebook groups for women in Sweden. The survey was conducted in April 2022. No information that could identify any individual participant was collected.

### Measurements

The participants’ level of PTG was measured using the Post-traumatic Growth Inventory-Short Form (PTGI-SF) [26]. This includes ten items describing five factors of PTG, to which the participants respond with their IPV relationship in mind, on a six-point Likert scale ranging from 1 (‘I did not experience this change as a result of my crisis’) to 6 (‘I experienced this change to a very great degree as a result of my crisis’). The Cronbach’s α from the sample was 0.81.

The survey included questions about whether the participants had received any formal help after they left their IPV relationship and, if so, what type of formal help they had received. The participants could choose one or more of the alternatives: individual therapy with a psychologist/therapist; supportive conversations; spiritual support; counselling; or others. They also indicated which help they thought had helped the most.

### Analysis

Attainment of PTG was examined by adapting the reference point for PTGI [11,12] to PTGI-SF (i.e. a total score of 40). The non-parametric test Kruskal–Wallis was used to compare different groups due to the violation of the normality assumption in our data [27]. Eta-squared was used for effect size.

## Results

The majority of the participants (69.3%; *n* = 115) reached or exceeded the pre-determined reference point for the attainment of PTG (i.e. a total score of 40). The mean score of PTG from the entire sample was 42.1 (SD = 8.85). The Kruskal–Wallis test showed that those who received formal help reached a significantly higher level of PTG than those who did not (Table I). The effect size was small (0.05). [28] Kruskal–Wallis test did not show a significant difference in PTG among the participants who indicated different types of formal help as the most helpful for them (Table I). The effect size was small (0.02) [28].

**Table I.** Level of PTG depending on the receipt and type of formal help.

Receipt of formal help	Mean (SD)	Test statistics	Type of formal help^a^	M (SD)	Test statistics
Those who received formal help (*n* = 119)	43.4 (8.3)	*χ*^2^ (1, 166) = 8.06**	Individual therapy^b^ (*n* = 50)	44.1 (9.2)	*χ*^2^ (2, 110) = 2.01 n.s.
			Supportive conversation^c^ (*n* = 40)	43.0 (7.0)	
			Counselling^d^ (*n* = 20)	45.4 (7.2)	
			Spiritual support^e^ (*n* = 0)	–	
Those who did not receive formal help (*n* = 47)	38.9 (9.4)		–		

** *p* < 0.01.

n.s. Not significant.

a Most of the participants received multiple types of formal help. What is presented here indicates the type of formal help that the participants indicated as the most helpful for them. Only 110 of the 119 respondents who received formal help indicated one type of help as the most helpful. Hence nine respondents were excluded from this question because they either could not indicate one single type of help that was more helpful than others or who said that informal help was more helpful than formal help.

b Conversation with psychologist or therapist, such as cognitive behavioural therapy, interpersonal therapy or psychodynamic therapy.

c Conversations at women’s shelters, group therapy or support groups.

d Conversations with a counsellor at a health centre, youth centre or likewise.

e Conversations with a religious leader or staff within a religious community.

SD: standard deviation.

## Discussion

This study showed that the majority of the participants attained PTG, which echoes with the recent emerging focus on the potential for the positive change among people with traumatic experiences [8–10]. This study expanded previous findings [11,12] that women who experienced IPV can reach PTG to a Swedish context.

The women who received formal help presented a higher level of PTG than those who did not. This corroborates previous research [12,14,15,19,21,23] by showing that formal help can help women in Sweden reach PTG after the experience of IPV. This is the first study to directly compare the level of PTG between those who received help and those who did not. Our findings highlight the important role that formal help has in facilitating PTG among women who experience IPV.

No significant difference was found in the level of PTG between the women who received different types of formal help. Previous have studies shown that different types of formal help can facilitate PTG [15,16,23,24], but did not investigate the potential differing effects of different types of formal help on PTG. This is the first study to directly compare the levels of PTG among people who found different types of formal help to be the most effective for them. These findings indicate that different types of formal help can work equally well as a tool to develop PTG after an IPV relationship. However, it should be noted that most women had received more than one type of formal help, hence which type of help contributed the most may differ from what they perceived as the most effective.

### Strengths and limitations

This is the first study to examine IPV and PTG among Swedish women and to compare the level of PTG depending on the receipt and type of formal help. The limitation is that PTGI-SF was translated into Swedish without a pre-validation procedure, which could have affected the results [26]. Also, the results are based on a homogenous sample of Swedish women and should therefore only be generalized to other populations with caution. Some demographic/background information that could have a significant role in women’s help-seeking behaviours/resources and PTG were not available in this study, such as socioeconomic status, ethnicity, religion, urbanicity, other accompanied trauma/difficulties, availability of role models, the level of post-traumatic stress disorder after IPV and the duration after IPV [11,6,29]. Hence it was not possible to investigate how these relevant factors might have influenced the study participants’ PTG.

### Future studies

This study focused only on formal help. Future studies could benefit from adding informal help, which women found important for their progress after an IPV relationship [13,15,30]. Also, future studies are recommended to recruit a more representative sample by, for example, sending the survey to a randomly chosen sample of all women in Sweden. In addition, men’s experiences of an IPV relationship should also receive research attention. We could also investigate more specifically which types of help contribute to the attainment of PTG.

## Conclusions

This study shows that most of the Swedish women who experienced an IPV relationship attained PTG. The finding also indicates that women who receive formal help attain PTG to a greater extent than women who do not. Different types of help appear to facilitate PTG to a similar extent, highlighting the importance of public health efforts to ensure an immediate connection between women who experienced IPV and available help of any kinds.
